# Physician-scientists’ perspectives on key factors, emotions and feelings about selecting and attending continuous professional development events: a mixed-method study

**DOI:** 10.1186/s12909-024-06015-8

**Published:** 2024-11-14

**Authors:** Stefano Sandrone, Terese Stenfors

**Affiliations:** 1 Department of Brain Sciences, Faculty of Medicine, Imperial College London, London, United Kingdom; 2 Department of Learning, Informatics, Management and Ethics, Karolinska Institutet, Stockholm, Sweden

**Keywords:** Clinician-scientist, Physician-scientist, Continuous Professional Development, CPD, Emotion, Feeling, Medical education, Lifelong learning

## Abstract

**Background:**

Almost 40% of the Nobel-Prize-winning discoveries in medicine are made by physician-scientists, who are a driving force in the evolving medical, academic and research landscape. However, their training has few defined milestones. To be effective clinicians, educators and researchers, they need to maintain and hone skills, often via continuous professional development (CPD) activities covering different domains. They have recurrently been described as an endangered species. Yet, warnings and recommendations across several decades did not stop the declining number of physician-scientists, which is now a chronic issue. This is further exacerbated by a lack of resources and support, especially after the COVID-19 pandemic.

**Methods:**

We administered a questionnaire called Positive and Negative Affect Schedule (PANAS-GEN) to get an initial emotional snapshot before performing individual semi-structured interviews with five physician-scientists in neurology working in the United Kingdom. We explored the key factors they balance before selecting CPD activities, along with their views on compulsory CPD events and assessments. We investigated their general feelings towards compulsory and non-compulsory CPD, how they felt the night before and the morning of the events, and the perceived consequences attending these have on their learning.

**Results:**

In our study, physician-scientists tend to choose training in their area of expertise but would enjoy exploring more if they had more time. The CPD choice was chiefly driven by speakers and topics, followed by learning needs. They disputed the utility of the current assessments, which are often seen as box-ticking exercises. While frustration, hostility and negative feelings were voiced for the compulsory ones, other CPD activities were welcomed with excitement, curiosity and a sense of adventure. Enthusiasm and excitement were felt the night before and the morning of the non-compulsory ones. CPD events were perceived to positively affect further learning, with the most immediate consequences being reading an article, networking or interacting with the speakers.

**Discussion:**

This is the first study exploring the key factors driving a group of physician-scientists while selecting CPD activities and investigating their feelings and emotions related to CPD attendance. More engaging and less box-ticking CPD should be on the cards, along with an adequate evaluation of these activities. It is essential to increase enthusiasm, which can facilitate engagement, and decrease frustration surrounding compulsory CPD activities. We still know too little about the role of emotions in learning, especially about CPD. Future studies should investigate the emotional side of learning across different career stages to restore the leaky pipeline and create a tailored environment with benefits for each of the three sides of the physician-scientist’s identity: the clinical, the research, and the academic.

**Supplementary Information:**

The online version contains supplementary material available at 10.1186/s12909-024-06015-8.

## Introduction

The expression *continuous professional development* (CPD) identifies the activities designed to stay current with the evolving landscape of a given profession and learn new skills. Although they can differ in form, objectives and delivery, within medical education they usually take the shape of seminars, conferences, short courses and workshops [15, 60]. While the specific regulations on compulsory CPD might change from one country to another, healthcare professionals globally attend these events to maintain competence [10, 21].

Physician-scientists (or clinician-scientists) have generally completed an MD and PhD [40], they ‘conduct independent scientific investigation in the laboratory, clinic, or other setting’ [46] and advance the medical field by translating basic research findings into bedside applications [30, 58]. They represent a key driving force in the biomedical landscape: almost 40% of Nobel Laureates in Physiology or Medicine [29] and about 70% of NIH institutional leadership and chief scientific officers of pharmaceutical companies [46] are physician-scientists. A broader definition also includes ‘basic, disease-oriented, patient-oriented, population-oriented, and prevention-oriented investigations’ [47, 49]. Many of them also have teaching commitments, adding a third hat beyond the dyad, thus becoming the ‘academic triad’ or ‘triple threat’ [34], although the physician-scientist label is the most frequently used definition.

The training of physician-scientists is long, relatively amorphous and has few defined milestones [4, 18]. It occurs ‘at a critical period of other life milestones, such as purchasing homes or expanding families’ and involves long years of training within ‘the growing complexities of both clinical and research documentation’ [34]. Moreover, physician-scientists invest significant time and effort in research while seeing patients in clinical practice [9] but they also need to maintain and hone skills to be effective clinicians, educators and researchers. This makes the training of such a professional figure unique in the healthcare landscape. On the one hand, designing CPD activities that help them meet the professional standard (potentially in a manner that is engaging and rewarding) should be a priority. It is essential to study the critical factors influencing participation and to improve the training. On the other hand, the declining number of physician-scientists has become a chronic issue [20, 57].

Such a professional figure has recurrently been described as a fragile ‘link in the medical research chain’ [49]: ‘endangered species’ in the *New England Journal of Medicine* in 1979 [62], ‘endangered and essential’ in *Science* twenty years after [47], ‘vanishing career’ [54] thirty years on, a profession to be saved forty years later yet still ‘endangered’ both before [29] and after the COVID-19 pandemic [46]. The proportion of US physicians doing research dropped to 1.6% in 2011 from 3.6% in 1982 [19, 40]. A similar trend has been seen worldwide, including in the UK [7]. In other countries such datasets have not been systematically recorded until a few years ago [56]. It has been estimated that only 1.5% of physicians conduct research as their primary profession [17, 41]. The lower income for physician-scientists vs clinical peers, difficulty balancing the two worlds, and lack of research resources and support are among the causes of this decline [7, 25].

Each decade saw strategies and recommendations to restore the leaky pipeline, from injecting more funding into the system to providing support at individual and programme levels [33, 59] and new guidelines on recruitment, retention and diversity [39, 52]. Unfortunately, such issues have been exacerbated by the increasingly sparse nature of NIH funding [14] and the pandemic [46], which seems to have left physician-scientists more stressed and less productive [31].

In this study, we explore physician-scientists’ perspectives on key factors, emotions and feelings about continuous professional development events. These are the overarching research questions that guided us while planning this educational study: which factors do physician-scientists balance before choosing their CPD activities? What are their feelings towards CPD? What do they think of CPD-related assessments? How do they perceive that CPD activities impact their learning?

## Methods

The format of individual interviews was considered the best one to explore participants’ views on the learning and the emotional aspects of CPD in their niche context. The semi-structured nature of the interviews allowed a mixture of rigour in following a plot and freedom to explore interesting topics that would emerge.

We contacted about 20 physician-scientists (PS) working at UK universities via email.

The inclusion criteria were the following:

- having completed a PhD;
- being a physician-scientist (clinician and researcher);
- having an academic affiliation (i.e., lecturer or assistant professor, senior lecturer or associate professor, reader, professor or honorary);
- working at one of the top 50 universities in the UK.

Five of them (1 female, 4 males) from the Department of Brain Sciences of the same university answered positively and participated in the study. At the time of the interviews, one participant was a senior lecturer and four participants were professors. The individual semi-structured interviews with participants lasted up to 40 minutes and took place remotely via Microsoft Teams. At the beginning of the call, the participants filled out the PANAS-GEN [61], a validated questionnaire administered live and online (via Microsoft Teams). It is a self-reported measure of affect and it took about three minutes to be completed. In this study, it was used to get an initial ‘snapshot’ from an emotional perspective. This questionnaire consists of twenty words that describe different feelings and emotions. The respondents stated to which degree they generally felt that way on a Likert scale of 5 points: 1 for ‘Very slightly or not at all’, 2 for ‘A little’, 3 for ‘Moderately’, 4 for ‘Quite a bit’ and 5 for ‘Extremely’. Within the PANAS-GEN, there are positive and negative affect questions. The positive affect (PA) questions are the following numbers: 1, 3, 5, 9, 10, 12, 14, 16, 17, 19. The negative affect (NA) ones are questions 2, 4, 6, 7, 8, 11, 13, 15, 18, & 20. Scores range from 10 to 50 for both sets of items [61].

The interview guide was specifically developed for this study (Supplementary File 1). This was the plot followed for the interviews:

*Continuous Professional Development (CPD) activities are part of your career. Let’s talk about them in more detail and from different perspectives. How do you choose a CPD training event? What are the key factors you balance before deciding?* (This was a scaled question, from 0 to 10, and covered cost, speakers, topic, who is attending, scheduling, venue, learning needs)*. Thinking about the topic of a CPD event, do you prefer to stay in your area of expertise or go outside topic-wise? How do you think learning should be assessed in the context of CPD? Is the presence of the assessment a factor you consider when you choose a CPD event? What is your general feeling towards CPD? How do you feel about compulsory CPD? Can you please tell me something to love about the CPD or something that makes you break up with the concept of CPD? Now imagine this is the evening before a new CPD event. How do you feel? Imagine it is the morning before the CPD event. Can you please tell me how you feel? And which emotion do you associate with participating in a professional development event? Have CPD activities led you to further learning/training? Thanks for taking the time to answer my questions. Do you have any questions for me?*

One of the authors, StS, conducted the interviews, which were automatically transcribed by the Microsoft Teams function and anonymised by StS. The transcriptions were manually proofread by the same author. No identifiable information was present in the raw data. The thematic analysis [13, 36] was conducted by StS and the codes were checked by TS. Both StS and TS are experienced educators and scholars. Two steps within the coding process were followed: open coding and axial coding [44], which led to identifying first-order and second-order codes. The codes were not defined *a priori* but emerged from the thematic analysis.

## Results

### PANAS-GEN scores, key factors selection-wise and themes

The participants had a total PANAS-GEN score between 17 and 24: PS2 and PS5 had 17, PS1 had 18, PS4 20 and PS3 24. They scored 39, 41, 45, 37 and 35 for PA and 21, 24, 21, 17 and 18 for NA (PS1 to PS5, Table 1). While answering the scaled question ‘What are the key factors you balance before deciding?’, speakers and topic scored an average of 8.9 on a scale from 0 (being not important at all) to 10 (being extremely important), followed by learning needs (7.1), venue (6.8), scheduling (6.7), other people attending (5.8) and cost (4) (Table 2). Three themes emerged from the thematic analyses of the answers to the interviews: ‘decisions’, ‘learning’, ‘emotions and feelings’.

**Table 1:**
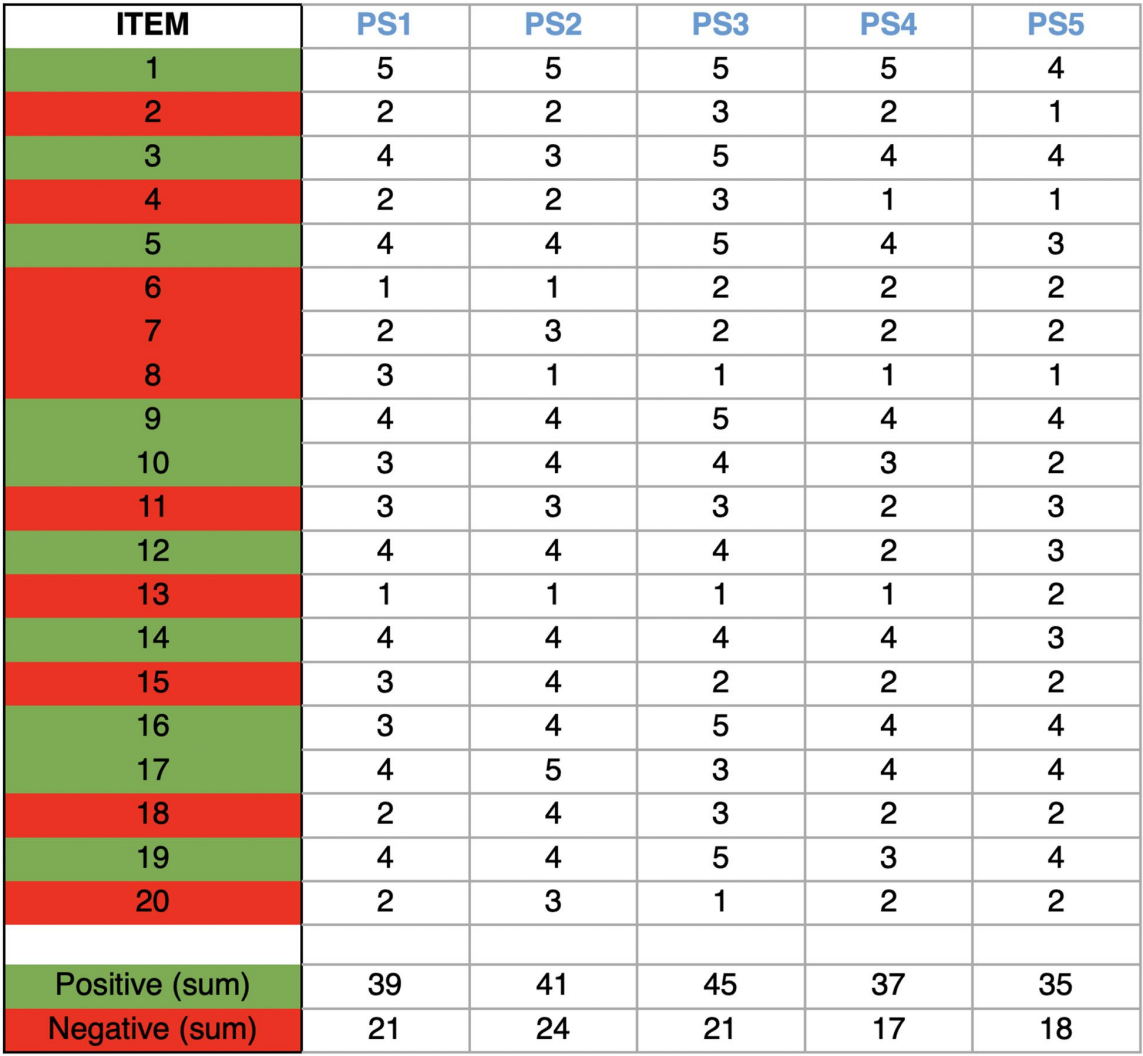
Item by item scores of the PANAS-GEN for positive affect and negative affect for each participant. The respondents stated to which degree they generally felt that way on a Likert scale of 5 points: 1 indicated ‘Very slightly or not at all’, 2 for ‘A little’, 3 for ‘Moderately’, 4 for ‘Quite a bit’ and 5 for ‘Extremely’

**Table 2:**
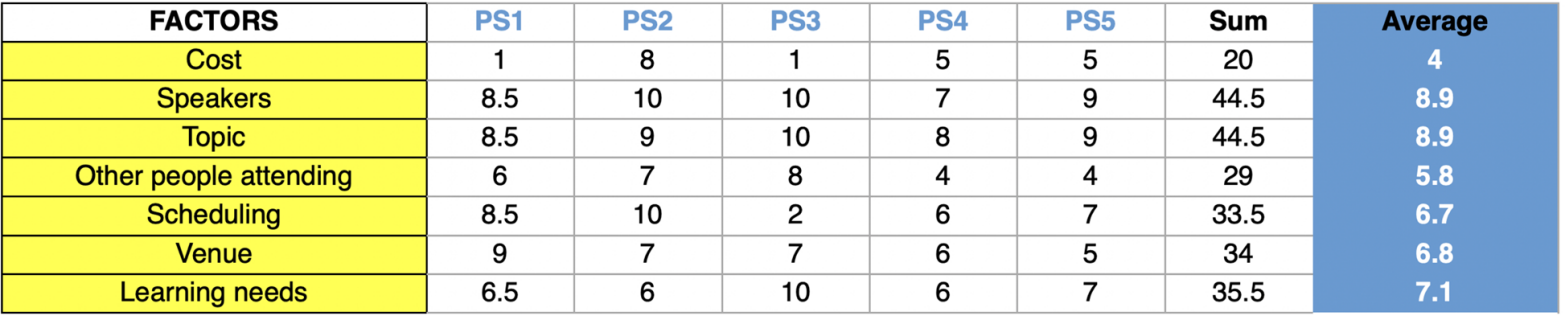
Participants’ answers to the scaled question (from 0 to 10): ‘*What are the key factors you balance before deciding?’*

### ‘Should I stay or should I go?’: Staying or leaving the area of expertise?

Topic-wise, the dominant preference was to stay in the area of expertise ‘to focus’ (PS2), ‘to extend that and to go into detail in the things I know about’ also because ‘my opinion is increasingly sought as an expert opinion’ (PS5). However, many interviewees expressed the willingness to explore other areas if they had more time (PS2, PS4) or to catch up when they ‘lost track’ of neurology-related topics they should be updated on (PS1). Only one participant, PS3, put the accent on systematically wanting to go outside to explore new areas: ‘based upon my interest at the time and a bit of randomness, if I see something which is particularly exciting out of the box, then I try to go’.

### Compulsory CPDs tick the boxes but kill enthusiasm (restored by the optional ones)

Bar one exception, feelings towards compulsory CPD were negative and sometimes even hostile. One of the interviewees said they felt ‘strongly against’ them and saw them as a ‘waste of time and money’ (PS1). Another interviewee, PS3, mentioned ‘hostile’ feelings towards the compulsory ones and ‘positive’ versus the non-compulsory ones. Then added: **‘**I'm quite against the structured ones (…) and I hate acronyms, CPD and all of this. But I do understand that it is important to keep track of the fact that people need to be engaged to develop their careers professionally without it becoming only a tick-the-box exercise’. PS5 declared: ‘I think the problem (…) is people tend to do the bare minimum and use it as a box-ticking exercise’. PS1 echoed it: ‘I really feel bad and I know that most of my colleagues feel bad as well because we are really put in a primary school frame of mind’. Only PS4 said ‘positive’ for both the compulsory and the optional ones: ‘it has to sit with the thought of having the chance to learn something new, update myself on current diagnostics or treatment pathways for conditions that I deal with. They are often occasions to get away from the regular day-to-day. And it's also often associated with networking with peers’. And continued: ‘on one hand, anything that is compulsory and mandatory (…) feels a little bit, let's say, negative, the perception in its definition of being compulsory or required has the feeling of being imposed onto. However, I think that it is very important, especially for clinical professionals (…) so, in a sense, there is a positive in this being compulsory (…) to make sure that everyone has access to (…) but I appreciate the rationale behind the choice of this being a compulsory thing. And, overall, I find it is good that it has to be done because actually clinicians know how important it is and want to do it’.

### ‘Odi et amo’ towards the concept of CPD

By digging deeper, an ‘odi et amo’ approach to CPD emerged. CPD was defined as ‘the formalisation of something that is a natural part of being a clinician and an academic clinician’ by PS5. But a mix of love and hate was reported for the ‘formalisation of it’, despite its intrinsic importance for the field of medicine, which is ever-evolving, being clearly acknowledged (PS5). PS4 loved the travelling involved in attending CPD events but also recognised the many opportunities virtual environments offer before adding that ‘as clinicians, we need to maintain standards, that's a must of clinical practice. We cannot practice without updating ourselves on the field. I don't think we can do without CPD. It's just that the term compulsory has a negative accent, in a way, because it almost makes us think of an imposition’. PS3 shared their love for ‘the content of the CPD’ and the hate and reluctance ‘to follow specific rules and guidance when CPD are compulsory’. PS2 emphasised the love for learning, but ‘certain aspects of the framework for CPD force you to make reflections that are unnecessary and can be frustrating (…) and to spend time keeping tabs of these activities because you have to, because you need to have certain professional requirements (…) It helps to have a recording tool and having a certification (…) but it burdens people who would do their CPD anyway because they do it, they just do it. It's part of their job, part of their role, part of their ethics. But on top of doing it, you have to document the time spent doing that. That's frustrating’. This was echoed by PS1, who claimed to love ‘to learn about the new developments in the field; for me, it is the most exciting thing to see’. However, PS1 also thought that ‘the CPD we do online is silly and we do it just to tick a box. Very little of that, less than 10% of that, is useful on a practical level or intellectually stimulating’.

### ‘I Gotta Feeling’: Feelings the night before and the morning of the event

Overall, the feelings before a CPD event, as described by the participants, were positive, albeit with different shades and intensity. From feeling well (in the words of PS1) to being full of excitement and anticipation (as framed by PS3), quite excited generally (PS5) or specifically ‘to go somewhere and meet some colleagues and listen to hopefully some good programs’ (PS4). But also a bit anxious if doing the CPD means talking in front of large crowds of people, then feeling energised after it: ‘you feel like you have been part of something good and that you're taking home a lot of ideas and a lot of energy’, with something that ‘really opened my brain so that it almost hurts (…) I can feel it, and this will be translated into some benefit to what I do’ (PS2). When the sun rises, the answers still point towards positive emotions: genuinely excited (PS5), excited, ‘similar to the evening before’ (PS3), rushed because of a delay but also ‘with that basic degree of enthusiasm that drives me’ (PS4). PS2 felt ‘rushed. Excited and keen. Yeah, generally slightly less anxious than the evening before, surely as long as I'm close to the venue’. ‘If it is one of those days when, before the appraisal, I have to do one of those tick-box exercises on the computer, I feel really bad and I feel I'm about to waste my time; if I know that there is a good speaker coming to give a lecture, I feel great. I really like doing that’, said PS1.

### Emotions associated with taking part in the CPD event and further learning

Generally, positive emotions, excitement and curiosity were associated with the physical or virtual act of participating in a CPD event. Sometimes, even a little bit of distrust has been mentioned, for example ‘if I have to go and listen to a talk where I'm not particularly convinced about the outcome of the research I see’ (PS4). ‘Satisfaction and something emotionally satisfying; rewarding intellectually’ were mentioned by PS1, ‘anxiety and excitement’ by PS2, ‘sense of adventure’ by PS3 and ‘excitement’ and a sense of interest by PS5. There was unanimous and reiterated agreement around the positive consequences of CPD on further learning: **‘**oh yeah, yeah, yeah, yeah, definitely yes’ (PS4); ‘yes, yes, yes, yes’ (PS3); ‘if I go to a particular talk or something that and find that a particular journal or something is interesting, then I'll go and read that article and follow it through’ (PS5). ‘I will go and check and do further research on it (…) I agree that happens a lot (PS1). ‘The typical example is this: you're not aware of the whole area of research or a single study, a single paper. At the next available opportunity, in a break or when you come home after CPD, you dig out that paper. It brings you up to speed on that and then you find something else (…) I think with almost all CPD, well, the good CPD, you come home with some seeds that you plant in your garden, and some of them will grow into plants and even trees, later on’ (PS2).

On an assessment-related note, four participants out of five said the CPD should not be assessed as physician-scientists needed to be ‘treated like adults’ (PS1), and there is no need to have an assessment. PS2 recognised that, didactically, assessments could play a role, but also highlighted that ‘asking people who are very busy professionally to take an exam after their activities will increase the time, the burden on them’ and ‘the chance they would hate it'. Instead of the assessment, according to PS3, the focus should be on ‘the achievements of the individual and how the individual is respected or not by their colleagues’. ‘I have a clinical appraisal and (…) the quality of my practise is assessed by my patients and by my colleagues’, said PS5. Only one participant, PS4, said CPD should be assessed.

## Discussion

Physician-scientists are fundamental assets in the medical, academic and research landscape, but they are endangered. They must maintain and extend their knowledge, skills and performance, as well as develop the skills required to be effective clinicians, educators and researchers [2], which is undoubtedly challenging. In the UK, CPD is mandatory for about ‘1.5 million individuals registered to work under 32 regulated titles’; 81% of those registered have to engage in reflective activities related to their learning, but only one in three should use a personal development plan (and 26% have no requirement to engage in peer-to-peer learning) [23]. Therefore, it is important to spotlight the factors they consider essential in relation to CPD events and the feelings and emotions about selecting and attending them.

Our findings suggest that physician-scientists tend to stay in their own area of expertise, especially as their opinion is increasingly sought as an expert one, although they would enjoy exploring more if they were allocated more time to do so. When they can select the CPD events to attend, their choice is chiefly driven by the speakers and topic, followed by learning needs, venue, scheduling, other people attending and cost. This aligns well with previous works showing that topical relevance is a critical aspect in influencing participation along with the quality of content, whereas time and cost are the main barriers [11]. Expense and travel time were identified as barriers to professional development in a study with 500 US clinicians, and optimising locations, reducing cost and allowing flexibility were suggested as potential solutions to ease the process [42]. Securing funding is reportedly one of the top three challenges that physician-scientists have to deal with, along with difficulty with the appointments and promotion process and the increasing burden of clinical activities [38]. However, the fact that cost was not mentioned as a major issue in this study might be linked to the interviewees being affiliated with a top university globally, hence potentially having access to more extensive resources. Yet cost and financial support are key aspects to consider while supporting the next generations of physician-scientists, also considering that those who self-identified as a race/ethnicity underrepresented in medicine and are between 40 and 49 are less likely to be satisfied with their CPD training and face higher obstacles [22].

Our interviewees recognised the importance of CPD (described as ‘the formalisation of something that is a natural part of being a clinician and an academic clinician’, PS5). Equally, they reiterated that having assessments can contribute to keeping a standard for the profession. However, they raised questions about the utility of the current assessments, often viewed as box-ticking exercises and perceived as non-engaging, not particularly useful and not conducive to treating them ‘like adults’ (PS1). Physicians’ engagement in CPD has been described as ‘fraught with challenges’: they are perceived as ‘impractical, decontextualised and check-box activities’ by participants [3]. The most frequent way of assessing CPD is written, including multiple-choice items, but a recent scoping review focusing on 130 reports showed that often the assessment is developed for research purposes rather than for the CPD activity itself [37], which is an aspect for improvement. While it is understandable that relying only on self-assessment is a difficult avenue to follow [53], more effort is needed to design engaging assessments. This should be paralleled with research on designing, developing and validating high-quality CPD assessments, balancing utility with physician-scientists’ satisfaction while responding to the needs of healthcare systems and societal expectations [45].

Yet too often there is limited insight into physician-scientists’ learning needs, such as little feedback data, and a tendency to engage in CPD activities ‘that were readily at hand—but not necessarily relevant’ and ‘to finding learning resources that might not be formally recognised for CPD credit’, as shown in a recent paper on twelve physicians from six different sub-specialities [3]. In the medium and long term, these aspects, the former more than the latter, might have detrimental consequences. ‘The problem with guidelines is people tend to do the bare minimum’, as epitomised by PS5: it is not just about ensuring the compulsory threshold is reached, but it is about the *what* and the *how*. Future studies should address how a professional competence system can be nurtured [3] in a more engaging manner. Very importantly, reducing bureaucracy might play a role in changing the perception of compulsory CPD.

In our study, we have also explored feelings and emotions in relation to CPD. Although healthcare practice and education are highly emotional endeavours, emotions, feelings and attitudes and their role in cognitive processes have been ignored for many decades [24, 26], and it is now time to shine a light on these aspects. The PANAS-GEN is used to quantify two dimensions of affect: positive affect and negative affect. Positive affect (PA) is the extent to which ‘a person feels active, alert, energised, engaged pleasurably, and able to concentrate’; low PA is characterised by sadness and lethargy [35]. Negative Affect (NA) is the ‘subjective distress that is present in a range of aversive mood states’ and ‘low NA is characterised by calmness and serenity’ [35, 61]. This scale has the advantage of being easy to administer. It has ‘excellent psychometric properties’, including good internal consistency [48] and is stable over time (i.e., over 8 weeks) [61]. It is reliable and widely used overall, yet it is still not extremely used in the postgraduate academic context. Here, we used it to capture an initial ‘snapshot’ from an emotional perspective. The PANAS-GEN is not a diagnostic instrument. Normative data in the UK collected from a sample of 1003 adults showed that the median PA was 32 and the mean was 31.31 [12]. In light of this, the PA scores collected in our samples were consistently higher than the normative sample cited in that study. Also the NA scores we collected were higher than those reported in the above-cited study, which calculated a median of 14 and a mean of 16 for NA [12]. While recognising we only had a single data point from a sample of five participants, we also acknowledge the value of including these scores in the manuscripts. They are rarely collected at the start of an interview within an academic setting, but these scores might set the basis for future explorations. Comparisons between different time points throughout the academic year can easily be made.

Moving from the quantitative to the qualitative side of the study, frustration, hostility and negative feelings have been voiced for compulsory CPD. In contrast, ‘the good ones’ were welcomed with excitement and anticipation, curiosity, a ‘sense of interest’ (PS5) or even a ‘sense of adventure’ (PS3). At a deeper analysis, it seems that the optional CPD events play a sort of a buffer role, or a compensatory mechanism, a re-balancing of the negative experience with the compulsory ones. In other words, the physician-scientists complete the compulsory ones because they must do them but have an overall negative (if not openly hostile) approach towards them and do little to hide their disagreement. Our participants seemed to enthusiastically embrace the ones they could choose, which tilt the balance towards a satisfactory level in the overarching equation. This might be the mechanism as, overall, clinicians are mainly satisfied with their ability to stay current via CPD training, as shown by an overwhelming 90% of 5926 respondents within the Association of American Medical Colleges' National Sample Survey of Physicians [22]. The enthusiasm and excitement all the participants felt the night before the CPD event and the morning itself (along with anxiety if they were to speak in front of a crowd) were notable. Enthusiasm and motivation can be key factors in facilitating CPD attendance [27, 32]. If this is paired with the fact that 315 first-year medical students surveyed at the beginning of their bachelor's program reported high levels of intrinsic and extrinsic motivation for ‘research, self-efficacy, perceptions of research, curiosity, and need for challenge’ [43], it seems all this enthusiasm is dissipated along the leaky pipeline. Students who have just joined medical school seem already motivated to do research; but why do fewer than two out of 100 continue becoming physician-scientists? This is a question for future studies. Cultivating a positive research culture and a proper support level for wannabe physician-scientists is essential. Educators hold an important role in sustaining that enthusiasm. The risk is to either have later, or lose forever, fundamental medical advancements.

Our findings show that CPD events positively affect further learning: ‘I think with almost all CPD, well, the good CPD, you come home with some seeds that you plant in your garden, and some of them will grow into plants and even trees, later on’ (PS2). The most reported consequence was reading a paper on the topic (‘then I’ll go and read that article’, PS5); the second was networking or interacting with the speakers. This aligns with previous research suggesting that knowledge is the most commonly reported measure of ‘impact’ [1]. Yet perhaps other dimensions should be captured, even if they seem more elusive or more difficult to be unequivocally linked to a specific CPD activity (‘practice change, skill, confidence, attitudes, career development, networking, user outcomes, intention to change’) [1]. Overall, there are gaps in the identification, quantification, pricing and analysis of cost outcomes: most studies compared a CPD activity against no intervention rather than a viable alternative intervention [10]. Many reports barely scrutinise the economic impact; when they do so, the cost lists have been defined as incomplete and lacking a satisfactory level of detail [10]. Growing and exploiting a network of colleagues is rarely a studied consequence, despite this being highly considered by participants, as we have seen in this study and as reported in twenty semi-structured in-depth interviews previously conducted [2]. The social learning process should be increasingly considered when designing and evaluating CPD for physician-scientists.

This is the first study exploring key factors driving a group of physician-scientists choosing CPD and their feelings and emotions related to CPD attendance, which is a remarkable strength. Very few works exist on emotions and feelings related to CPD, and we still know little about the role of emotions in learning. To improve medical education, we must include emotions in the exploration [5, 8]. We need to go beyond the paucity of papers on how physician-scientists think, feel and reason about the competing demands on their time and effort, directly affecting their own success and the organisational success [51]. Another strength of this work is that all the physician-scientists surveyed and interviewed work in the same discipline, neurology, and in the same country, the UK. In light of the Information Power model and given the clear aims of this study, the sample specificity, the quality of the dialogue and the analysis strategy, the sample size is acceptable [28, 55].

Limitations-wise, only one female physician-scientist was interviewed; unfortunately, this is symptomatic of a broader problem, which is linked to the low numbers of female academics and medical doctors enrolled in universities worldwide. Historically, women are underrepresented in the physician-scientist pool [4, 6]. Some studies shed light on the reasons behind this [16], but more should be done. Overall, few works exist on factors towards CPD selection and attendance and on the concept of *identity* among physician-scientists, and there is ‘a lack of evidence about the specific determinants of clinician-scientist professional identity development’ [50].

In conclusion, it is essential to increase enthusiasm and decrease the sense of frustration surrounding compulsory CPD activities. More engaging and less box-ticking CPD should be on the cards. Future studies should investigate the emotional side of learning across different career stages to restore the leaky pipeline and create a tailored environment. This can, in turn, bring benefits to each of the three sides of the physician-scientist’s identity: the clinical, the research and the academic.

## Supplementary Information

Supplementary Material 1.

## Data Availability

The data analysed in this study are available upon reasonable request.

## References

[1] Allen LM, Palermo C, Armstrong E, Hay M. Categorising the broad impacts of continuing professional development: a scoping review. Med Educ. 2019;53(11):1087–99. 31396999 10.1111/medu.13922

[2] Allen LM, Hay M, Armstrong E, Palermo C. Applying a social theory of learning to explain the possible impacts of continuing professional development (CPD) programs. Med Teach. 2020;42(10):1140–7. 32706608 10.1080/0142159X.2020.1795097

[3] Allen LM, Balmer D, Varpio L. Physicians’ lifelong learning journeys: a narrative analysis of continuing professional development struggles. Med Educ. 2024;58(9):1086–96. 38605442 10.1111/medu.15375

[4] Andrews NC. The other physician-scientist problem: where have all the young girls gone? Nat Med. 2002;8(5):439–41. 11984578 10.1038/nm0502-439

[5] Artino AR Jr, Durning SJ. It’s time to explore the role of emotion in medical students’ learning. Acad Med. 2011;86(3):275. 10.1097/ACM.0b013e318208437f 21346426

[6] Brown NJ. Promoting the success of women and minority physician-scientists in academic medicine: a dean’s perspective. J Clin Invest. 2020;130(12):6201–3. 33021966 10.1172/JCI144526 PMC7685745

[7] Chandrapalan S, Phillips C, Newbery N, Logan S, Arasaradnam R. Research activity among physicians in the United Kingdom: results from the Royal College of Physicians Census 2022. Clin Med. 2023;23(6):637–40. 10.7861/clinmed.2023-0388 PMC11046586 38052464

[8] Cleland J, Durning SJ, editors. Researching medical education. Chichester: John Wiley & Sons; 2022.

[9] Cola PA, Wang Y. Discovering Factors that influence physician scientist success in academic medical centers. Qual Health Res. 2022;32(10):1433–46. 35737579 10.1177/10497323221108639

[10] Cook DA, Stephenson CR, Wilkinson JM, Maloney S, Thomas BL, Prokop LJ, Foo J. Costs and economic impacts of physician continuous professional development: a systematic scoping review. Acad Med. 2022;97(1):152–61. 34432716 10.1097/ACM.0000000000004370

[11] Cook DA, Price DW, Wittich CM, West CP, Blachman MJ. Factors influencing physicians’ selection of continuous professional development activities: a cross-specialty national survey. J Contin Educ Health Prof. 2017;37(3):154–60. 28767542 10.1097/CEH.0000000000000163

[12] Crawford JR, Henry JD. The Positive and Negative Affect Schedule (PANAS): Construct validity, measurement properties and normative data in a large non-clinical sample. Br J Clin Psychol. 2004;43(3):245–65. 15333231 10.1348/0144665031752934

[13] Dawadi S. Thematic analysis approach: A step by step guide for ELT research practitioners. J NELTA. 2021;25(1–2):62–71.

[14] Daye D, Patel CB, Ahn J, Nguyen FT. Challenges and opportunities for reinvigorating the physician-scientist pipeline. J Clin Invest. 2015;125(3):883–7. 25689260 10.1172/JCI80933 PMC4362227

[15] Davis D, O’Brien MA, Freemantle N, Wolf FM, Mazmanian P, Taylor-Vaisey A. Impact of formal continuing medical education: do conferences, workshops, rounds, and other traditional continuing education activities change physician behavior or health care outcomes? JAMA. 1999;282(9):867–74. 10478694 10.1001/jama.282.9.867

[16] Edmunds LD, Ovseiko PV, Shepperd S, Greenhalgh T, Frith P, Roberts NW, Pololi LH, Buchan AM. Why do women choose or reject careers in academic medicine? A narrative review of empirical evidence. Lancet. 2016;388(10062):2948–58. 27105721 10.1016/S0140-6736(15)01091-0

[17] Ellaithy A, Narayanan NS. Opinion and Special Articles: Mentoring in neurology: Where are the clinician-scientists? Is residency to blame? Neurology. 2019;92(24):1159–62. 31182513 10.1212/WNL.0000000000007657 PMC6598794

[18] Gallagher EJ, Conlin PR, Kazmierczak BI, Vyas JM, Ajijola OA, Kontos CD, Baiocchi RA, Rhee KY, Hu PJ, Isales CM, Williams CS. Is it time to reduce the length of postgraduate training for physician-scientists in internal medicine? JCI Insight. 2024;9(10). 10.1172/jci.insight.178214 PMC11141926 38775155

[19] Garrison HH, Deschamps AM. NIH research funding and early career physician scientists: continuing challenges in the 21st century. FASEB J. 2014;28(3):1049–58. 24297696 10.1096/fj.13-241687 PMC3929670

[20] Gill GN. The end of the physician-scientist? The American Scholar. 1984:353-68.

[21] Hilty DM, Liu HY, Stubbe D, Teshima J. Defining professional development in medicine, psychiatry, and allied fields. Psychiatr Clin North Am. 2019;42(3):337–56. 31358116 10.1016/j.psc.2019.04.001

[22] Jayas A, Andriole DA, Grbic D, Hu X, Dill M, Howley LD. Physicians’ continuing medical education activities and satisfaction with their ability to stay current in medical information and practice: A cross-sectional study. Health Sci Rep. 2023;6(2):e1110. 36789399 10.1002/hsr2.1110 PMC9918722

[23] Karas M, Sheen NJ, North RV, Ryan B, Bullock A. Continuing professional development requirements for UK health professionals: a scoping review. BMJ Open. 2020;10(3):e032781. 10.1136/bmjopen-2019-032781 PMC7066625 32161156

[24] LeBlanc VR, McConnell MM, Monteiro SD. Predictable chaos: a review of the effects of emotions on attention, memory and decision making. Adv Health Sci Educ Theory Pract. 2015;20:265–82. 10.1007/s10459-014-9516-6 24903583

[25] Lingard L, Zhang P, Strong M, Steele M, Yoo J, Lewis J. Strategies for supporting physician–scientists in faculty roles: a narrative review with key informant consultations. Acad Med. 2017;92(10):1421–8. 28795977 10.1097/ACM.0000000000001868

[26] Louwen C, Reidlinger D, Milne N. Profiling health professionals’ personality traits, behaviour styles and emotional intelligence: a systematic review. BMC Med Educ. 2023;23(1):120. 36803372 10.1186/s12909-023-04003-y PMC9938999

[27] Macdougall C, Epstein M, Highet L. Continuing professional development: putting the learner back at the centre. Arch Dis Childhood-Education Pract. 2017;102(5):249–53. 10.1136/archdischild-2016-310864 28302733

[28] Malterud K, Siersma VD, Guassora AD. Sample size in qualitative interview studies: guided by information power. Qual Health Res. 2016;26(13):1753–60. 26613970 10.1177/1049732315617444

[29] Jain MK, Cheung VG, Utz PJ, Kobilka BK, Yamada T, Lefkowitz R. Saving the endangered physician-scientist—a plan for accelerating medical breakthroughs. N Engl J Med. 2019;381(5):399–402. 31365796 10.1056/NEJMp1904482

[30] Kosik RO, Tran DT, Fan AP, Mandell GA, Tarng DC, Hsu HS, Chen YS, Su TP, Wang SJ, Chiu AW, Lee CH. Physician scientist training in the United States: a survey of the current literature. Eval Health Prof. 2016;39(1):3–20. 24686746 10.1177/0163278714527290

[31] Kwan JM, Noch E, Qiu Y, Toubat O, Christophers B, Azzopardi S, Gilmer G, Wiedmeier JE, Daye D. The impact of COVID-19 on physician-scientist trainees and faculty in the United States: A national survey. Acad Med. 2022;97(10):1536–45. 35921163 10.1097/ACM.0000000000004802 PMC9547818

[32] Lee NJ. An evaluation of CPD learning and impact upon positive practice change. Nurs Educ Today. 2011;31(4):390–5. 10.1016/j.nedt.2010.07.012 21129826

[33] Ley TJ, Rosenberg LE. The physician-scientist career pipeline in 2005: build it, and they will come. JAMA. 2005;294(11):1343–51. 16174692 10.1001/jama.294.11.1343

[34] Lin DJ, Cudkowicz ME, Cho TA. Opinion and Special Articles: Challenges and opportunities in defining career identity in academic neurology. Neurology. 2018;91(14):670–2. 30275123 10.1212/WNL.0000000000006284

[35] Medvedev ON, Roemer A, Krägeloh CU, Sandham MH, Siegert RJ. Enhancing the precision of the Positive and Negative Affect Schedule (PANAS) using Rasch analysis. Curr Psychol. 2023;42(2):1554–63.

[36] Morgan H. Conducting a qualitative document analysis. Qual Rep. 2022;27(1):64–77.

[37] Marceau M, Lachiver ÉV, Lambert D, Daoust J, Dion V, Langlois MF, McConnell M, Thomas A, St-Onge C. Assessment practices in continuing professional development activities in health professions: A scoping review. J Contin Educ Health Prof. 2024;44(2):81–9. 37490015 10.1097/CEH.0000000000000507

[38] McKinney RE Jr. The daunting career of the physician–investigator. Acad Med. 2017;92(10):1368–70. 28767494 10.1097/ACM.0000000000001869

[39] Milewicz DM, Lorenz RG, Dermody TS, Brass LF. Rescuing the physician-scientist workforce: the time for action is now. J Clin Invest. 2015;125(10):3742–7. 26426074 10.1172/JCI84170 PMC4607120

[40] Morel PA, Ross G. The physician scientist: balancing clinical and research duties. Nat Immunol. 2014;15(12):1092–4. 25396341 10.1038/ni.3010

[41] National Institutes of Health (NIH). Physician-Scientist Workforce (PSW) Working Group Report. NIH Website. Available at: acd.od.nih.gov/documents/reports/PSW_Report_ACD_06042014.pdf. Accessed 8 Oct 2024.

[42] O’Brien Pott M, Blanshan AS, Huneke KM, Baasch Thomas BL, Cook DA. Barriers to identifying and obtaining CME: a national survey of physicians, nurse practitioners and physician assistants. BMC Med Educ. 2021;21:1–8. 33740962 10.1186/s12909-021-02595-x PMC7975233

[43] Ommering BW, van Blankenstein FM, Waaijer CJ, Dekker FW. Future physician-scientists: could we catch them young? Factors influencing intrinsic and extrinsic motivation for research among first-year medical students. Perspect Med Educ. 2018;7:248–55. 30006870 10.1007/s40037-018-0440-y PMC6086821

[44] Peel KL. A beginner’s guide to applied educational research using thematic analysis. Prac Assess Res Eval. 2020;25(1):2.

[45] Prior Filipe H, Gwen Mack H. Once upon a time there was CME, and then…“Expanding the voices in CME-CPD.” J CME. 2023;12(1):2270280. 37937264 10.1080/28338073.2023.2270280 PMC10627041

[46] Rao RC, Dlouhy BJ, Capell BC, Akeju O. The endangered physician-scientist and COVID-19. Cell Rep Med. 2021;2(2):100190. 33521693 10.1016/j.xcrm.2021.100190 PMC7832285

[47] Rosenberg LE. Physician-scientists—endangered and essential. Science. 1999;283(5400):331–2. 9925491 10.1126/science.283.5400.331

[48] Roemer A, Medvedev ON. Positive and Negative Affect Schedule (PANAS). In: Handbook of assessment in mindfulness research 2023 (pp. 1-11). Cham: Springer International Publishing.

[49] Rosenberg LE. The physician-scientist: an essential—and fragile—link in the medical research chain. J Clin Invest. 1999;103(12):1621–6. 10377167 10.1172/JCI7304 PMC408393

[50] Rosenblum ND, Kluijtmans M, Ten Cate O. Professional identity formation and the clinician–scientist: a paradigm for a clinical career combining two distinct disciplines. Acad Med. 2016;91(12):1612–7. 27254011 10.1097/ACM.0000000000001252

[51] Rubio DM, Primack BA, Switzer GE, Bryce CL, Seltzer DL, Kapoor WN. A comprehensive career-success model for physician–scientists. Acad Med. 2011;86(12):1571–6. 22030759 10.1097/ACM.0b013e31823592fd PMC3228877

[52] Salata RA, Geraci MW, Rockey DC, Blanchard M, Brown NJ, Cardinal LJ, Garcia M, Madaio MP, Marsh JD, Todd RF III. US physician-scientist workforce in the 21st century: recommendations to attract and sustain the pipeline. Acad Med. 2018;93(4):565–73. 28991849 10.1097/ACM.0000000000001950 PMC5882605

[53] Sargeant J, Wong BM, Campbell CM. CPD of the future: a partnership between quality improvement and competency-based education. Med Educ. 2018;52(1):125–35. 28984354 10.1111/medu.13407

[54] Schafer AI. The vanishing physician-scientist? Transl Res. 2010;155(1):1–2. 20004354 10.1016/j.trsl.2009.09.006 PMC2796254

[55] Stenfors T, Kajamaa A, Bennett D. How to… assess the quality of qualitative research. Clin Teach. 2020;17(6):596–9. 32790137 10.1111/tct.13242

[56] Traill CL, Januszewski AS, Larkins R, Keech AC, Jenkins AJ. Time to research Australian physician-researchers. Intern Med J. 2016;46(5):550–8. 26909676 10.1111/imj.13043

[57] Zemlo TR, Garrison HH, Partridge NC, Ley TJ. The physician-scientist: career issues and challenges at the year 2000. FASEB J. 2000;14(2):221–30. 10657979 10.1096/fasebj.14.2.221

[58] Utz PJ, Jain MK, Cheung VG, Kobilka BK, Lefkowitz R, Yamada T, Dzau VJ. Translating science to medicine: the case for physician-scientists. Sci Transl Med. 2022;14(632):eabg7852. 35171650 10.1126/scitranslmed.abg7852

[59] Varki A, Rosenberg LE. Emerging opportunities and career paths for the young physician-scientist. Nat Med. 2002;8(5):437–9. 11984577 10.1038/nm0502-437

[60] Vinas EK, Schroedl CJ, Rayburn WF. Advancing academic continuing medical education/continuing professional development: adapting a classical framework to address contemporary challenges. J Contin Educ Health Prof. 2020;40(2):120–4. 32167961 10.1097/CEH.0000000000000286

[61] Watson D, Clark LA, Tellegen A. Development and validation of brief measures of positive and negative affect: the PANAS scales. J Pers Soc Psychol. 1988;54(6):1063. 3397865 10.1037//0022-3514.54.6.1063

[62] Wyngaarden JB. The clinical investigator as an endangered species. N Engl J Med. 1979;301(23):1254–9. 503128 10.1056/NEJM197912063012303

