# Supplementary material for: Physician-scientists’ perspectives on key factors, emotions and feelings about selecting and attending continuous professional development events: a mixed-method study
Source: BMC Med Educ. 2024 Nov 14;24:1306. doi: 10.1186/s12909-024-06015-8 (PMC11566134; doi:10.1186/s12909-024-06015-8)
Supplement: Supplementary file 1 — Supplementary Material 1. [file 12909_2024_6015_MOESM1_ESM.pdf]

Supplementary File 1

Continuous Professional Development (CPD) activities are part of your career.

Let's talk about them in more detail and from different perspectives.

How do you choose a CPD training event?

What are the key factors you balance before deciding? (This is a scaled question, from 0 to 10, covering cost, speakers, topic, who is attending, scheduling, venue and learning needs).

Thinking about the topic of a CPD event, do you prefer to stay in your area of expertise or go outside topic-wise?

How do you think learning should be assessed in the context of CPD?

Is the presence of the assessment a factor you consider when you choose a CPD event?

What is your general feeling towards CPD?

How do you feel about compulsory CPD?

Can you please tell me something to love about the CPD or something that makes you break up with the concept of CPD?

Now imagine this is the evening before a new CPD event. How do you feel?

Imagine it is the morning before the CPD event. Can you please tell me how you feel?

And which emotion do you associate with participating in a professional development event?

Have CPD activities led you to further learning/training?

Thanks for taking the time to answer my questions. Do you have any questions for me?
